# Visualizing Hyperactivation in Neurodegeneration Based on Prefrontal Oxygenation: A Comparative Study of Mild Alzheimer's Disease, Mild Cognitive Impairment, and Healthy Controls

**DOI:** 10.3389/fnagi.2017.00287

**Published:** 2017-09-01

**Authors:** Kah Hui Yap, Wei Chun Ung, Esther G. M. Ebenezer, Nadira Nordin, Pui See Chin, Sandheep Sugathan, Sook Ching Chan, Hung Loong Yip, Masashi Kiguchi, Tong Boon Tang

**Affiliations:** ^1^Medicine Based Department, Royal College of Medicine Perak, Universiti Kuala Lumpur Kuala Lumpur, Malaysia; ^2^Centre for Intelligent Signal and Imaging Research, Universiti Teknologi Petronas Seri Iskandar, Malaysia; ^3^Community Based Department, Royal College of Medicine Perak, Universiti Kuala Lumpur Kuala Lumpur, Malaysia; ^4^Research & Development Group, Hitachi Ltd. Tokyo, Japan

**Keywords:** mild Alzheimer's disease, mild cognitive impairment, functional near-infrared spectroscopy, semantic verbal fluency task, prefrontal hemoglobin oxygenation

## Abstract

**Background:** Cognitive performance is relatively well preserved during early cognitive impairment owing to compensatory mechanisms.

**Methods:** We explored functional near-infrared spectroscopy (fNIRS) alongside a semantic verbal fluency task (SVFT) to investigate any compensation exhibited by the prefrontal cortex (PFC) in Mild Cognitive Impairment (MCI) and mild Alzheimer's disease (AD). In addition, a group of healthy controls (HC) was studied. A total of 61 volunteers (31 HC, 12 patients with MCI and 18 patients with mild AD) took part in the present study.

**Results:** Although not statistically significant, MCI exhibited a greater mean activation of both the right and left PFC, followed by HC and mild AD. Analysis showed that in the left PFC, the time taken for HC to achieve the activation level was shorter than MCI and mild AD (*p* = 0.0047 and 0.0498, respectively); in the right PFC, mild AD took a longer time to achieve the activation level than HC and MCI (*p* = 0.0469 and 0.0335, respectively); in the right PFC, HC, and MCI demonstrated a steeper slope compared to mild AD (*p* = 0.0432 and 0. 0107, respectively). The results were, however, not significant when corrected by the Bonferroni-Holm method. There was also found to be a moderately positive correlation (*R* = 0.5886) between the oxygenation levels in the left PFC and a clinical measure [Mini-Mental State Examination (MMSE) score] in MCI subjects uniquely.

**Discussion:** The hyperactivation in MCI coupled with a better SVFT performance may suggest neural compensation, although it is not known to what degree hyperactivation manifests as a potential indicator of compensatory mechanisms. However, hypoactivation plus a poorer SVFT performance in mild AD might indicate an inability to compensate due to the degree of structural impairment.

**Conclusion:** Consistent with the scaffolding theory of aging and cognition, the task-elicited hyperactivation in MCI might reflect the presence of compensatory mechanisms and hypoactivation in mild AD could reflect an inability to compensate. Future studies will investigate the fNIRS parameters with a larger sample size, and their validity as prognostic biomarkers of neurodegeneration.

## Introduction

The multiple dementia subtypes are associated with unique symptom patterns and brain abnormalities. Alzheimer's disease (AD) is a chronic progressive neurodegenerative brain disease that can occur in middle or old age. It represents the most common cause of dementia, accounting for 60–80% of cases (Alzheimer's Association, 2016). It causes increasing impairment in a range of cognitive functions that include memory, mood, reasoning, language, self-management, and behavior (Karantzoulis and Galvin, 2014). Mild cognitive impairment (MCI) is an intermediate state of clinical impairment, where the individuals affected have cognitive symptoms of a mild nature that are disproportionate to their age and education, while not meeting the criteria for dementia or AD (Petersen, 2009). Patients with MCI tend to progress toward developing AD at a rate of ~15% per year (Gauthier et al., 2006).

Magnetic resonance imaging (MRI) studies have demonstrated that deficits found in patients with AD are associated with volumetric changes in the prefrontal cortex (PFC) (Salat et al., 2001; McNab and Klingberg, 2008; Zanto et al., 2011). It has been suggested that the cognitive decline related to normal aging is attributable to a reduction in white matter rather than gray matter (Marner et al., 2003). In contrast, significant loss of gray matter, which is composed of cortical neurons and glia has been seen in AD, and leads to a reduced neuronal activation in the PFC of AD when compared with HC (Yankner et al., 2008). MCI is positioned between mild AD and normal cognitive aging (Sperling, 2007). While age-related regional volume loss is apparent and widespread in normal cognitive aging, a unique pattern of structural vulnerability, reflected in differential volume loss in specific regions, has been identified in patients with MCI (Driscoll et al., 2009). Also, patients with AD perform more poorly in semantic tasks associated with compromised activation in the left PFC when assessed using functional MRI (fMRI) (Johnson et al., 2000). The hippocampus is among the first non-cortical regions affected by AD-related neurodegeneration; hippocampal atrophy has been associated with early memory decline in MCI and AD (Barnes et al., 2007; Shi et al., 2009; Nho et al., 2012). Several previous studies of MCI patients and healthy older adults reported reduced activation in the hippocampus and PFC of the former (Johnson et al., 2006; Dannhauser et al., 2008; Mandzia et al., 2009). These results suggest that AD may be characterized by reduced brain activation due to the pathological changes associated with it.

One key element influencing these early changes is neural compensation. The capacity for neural compensation is inversely proportional to the severity of neurodegeneration (Price and Friston, 2002). As neural damage worsens, both cognitive processing efficiency and capacity become impeded. This affects neuronal recruitment capacity, reduces compensation capacities, and ultimately results in poorer performance (Scarmeas et al., 2003). As an example, transcranial magnetic stimulation has been shown to induce compensatory activation associated with information retrieval when applied to either the right or left PFC of patients with severe cognitive impairment. However, similar activation was only observed when applied to the left PFC of healthy individuals. These data suggest that right PFC recruitment acts as one of the functional compensatory mechanisms in cognitively impaired individuals (Cotelli et al., 2008). In addition, neural compensation prolonged the period of MCI and delayed progression to AD (Baazaoui et al., 2017), implying that such compensatory mechanisms may play a more crucial role in MCI than in AD. In the present study, the aim was to investigate the difference between MCI and AD using functional near-infrared spectroscopy (fNIRS) to identify hyperactivation as a potential indicator of the occurrence of compensation.

fNIRS is an emerging technology in neuroimaging that has been increasingly employed in the past 20 years (Ferrari and Quaresima, 2012). It uses an optical window where the head scalp is almost transparent to near infrared light at wavelengths of 700–900 nm, and the fact that oxygenated- and deoxygenated-hemoglobin (oxy-Hb and deoxy-Hb) are strong absorbers of light (Heinzel et al., 2013). Based on the concept of neurovascular coupling, neuronal activities are measured by fNIRS as changes in oxy-Hb and deoxy-Hb concentrations using the Modified Beer-Lambert Law. fNIRS has, so far, been applied to many psychiatric disorders, for example, to differentiate depression, bipolar disorder, and schizophrenia (Fallgatter et al., 1997; Arai et al., 2006; Takizawa et al., 2014). The technique has a good temporal resolution (~1 ms) and a reasonable spatial resolution (~1 cm). In addition, it has several advantages over the more widely-used fMRI, including lower costs, portability, and low levels of subject constraint (Ehlis et al., 2014). It has been demonstrated that fNIRS readings are highly correlated with fMRI with respect to measuring cognitive tasks (Cui et al., 2011). More specifically, multiple fNIRS-fMRI studies have consistently shown increases in oxy-Hb in the left PFC during semantic verbal fluency tasks (SVFT) (Fallgatter et al., 1997; Heinzel et al., 2013; Wagner et al., 2014; Gutierrez-Sigut et al., 2015). So, fNIRS represents a potential alternative technique to fMRI for the investigation of the differences in neuronal activities between individuals with differing cognitive impairments.

fNIRS has been used in previous studies into AD and MCI. Patients with AD were identified to have lower activation of the PFC compared to healthy controls (HC) during both letter and SVFT, while HC performed better in the tasks. Differences in hemodynamic responses were predominantly found in the left hemisphere, supporting the idea that good performances in verbal fluency tasks are associated with higher left hemisphere activation, predominantly (Fallgatter et al., 1997; Arai et al., 2006; Herrmann et al., 2008). This poorer performance in patients with AD has been attributed to the loss of hemispheric asymmetry rather than to the level of PFC activation alone (Fallgatter et al., 1997). The effect of hemispheric asymmetry or lateralization plays a more crucial role in MCI. The rightward shift of frontal activations in the MCI group might reflect the presence of cortical reorganization; the recruitment of the right PFC has been suggested to compensate the loss in the left PFC (Yeung et al., 2016). Another study has shown that compared to HC, patients with AD demonstrated lower activation of the frontal and bilateral parietal areas, while patients with MCI have lower right parietal activation (Arai et al., 2006). These results suggest that more detailed studies into neural compensatory mechanisms are warranted.

In the present study, fNIRS was used with a wide coverage of the PFC to investigate hyper/ hypoactivation in MCI, mild AD, and HC. The PFC was selected as the region of interest as the PFC is not only involved in semantic memory, but is also accessible by fNIRS, which can penetrate brain tissue up to a depth of 5 cm in the cortical region (Ranger et al., 2011). SVFT was selected as the cognitive activation task based on previous studies of semantic memory (Fallgatter et al., 1997; Thompson-Schill et al., 1997; Perry et al., 2000; Grossman et al., 2002; Heinzel et al., 2013; Wagner et al., 2014; Gutierrez-Sigut et al., 2015; Yeung et al., 2016). In accordance with the compensation theory (Yankner et al., 2008), it was expected that neural compensation would be observed in MCI, but not in AD during the tasks. Patients with MCI suffer from a relatively small degree of neurodegeneration compared to patients with AD, therefore, it was predicted that MCI patients would be able to activate neural compensation, as manifested by brain hyperactivation, in order to maintain task performance. As patients with AD suffer from more severe neuronal damage, to the extent that their neural compensation abilities might be compromised, it was thought that hypoactivation would be observed instead. We aimed to investigate to what degree hyperactivation, as a potential indicator for compensation, manifests in MCI and to what degree it is compromised in AD. Our first hypothesis was that during SVFT, subjects with normal cognitive aging (the HC group) would perform better, followed by MCI and AD. Secondly, we expected hyperactivation and hypoactivation in the PFC of MCI and AD, respectively, when subjects were tested with a semantic memory task.

## Methods

### Participants

We recruited participants, who were right-handed and able to converse in English, through a local dementia day-care center as well as from the local community where English was the common medium of instruction in the past. Patients with MCI and patients with mild AD were recruited through purposive sampling with group-specific inclusion criteria. This was followed by the recruitment of HC who were age (± 2 years), gender- and education-matched. We assessed the participants, ruling out anyone with a psychiatric disorder. Additionally, neurological disorders, including other forms of dementia were excluded. Assessments were performed by a psychiatrist using an evaluation of medical history, including careful examination of the course of progression, the relative salience of cognitive, behavioral and physical symptoms and signs, and patterns of cognitive impairment. Additionally, mental state was examined using a Mini-Mental State Examination (MMSE), which is a 30-point questionnaire providing a quantitative measure of cognitive status or cognitive impairment (Folstein et al., 1975). Other exclusion criteria were other medical diagnoses affecting cognitive functioning, including kidney failure, stroke, known lesions, and any history of significant trauma. The study protocol was approved by the Medical Research Ethics Committee of the University of Kuala Lumpur (Approval no.: 2015/032). All participants were briefed about the nature of the experimental procedures prior to providing demographic information and written informed consent in accordance with the Declaration of Helsinki. All tests and experiments were completed on the same day, with a short break between the test and the experiment. Both healthy participants and patients were remunerated for their participation.

### Clinical measures

We used the Clinical Dementia Rating (CDR), an observer rating scale designed to rate the severity of dementia (Morris, 1993), for the diagnosis of dementia and group allocation. CDR scores of 0, 0.5, and 1 were assigned to HC, MCI, and mild AD, respectively; participants with CDR scores of 2 (moderate) and 3 (severe) were excluded, as this study focused on MCI and mild AD. In addition, we assessed and assigned each participant an MMSE score. CDR has a moderate to high inter-rater reliability of 0.62 (Rockwood et al., 2000). MMSE has high inter-rater reliability, ranging between 0.82 and 0.91 (Magni et al., 1996). We matched the HC to those of the combined sample of patients according to age, sex, and education.

### fNIRS technology

**Figure 1:**
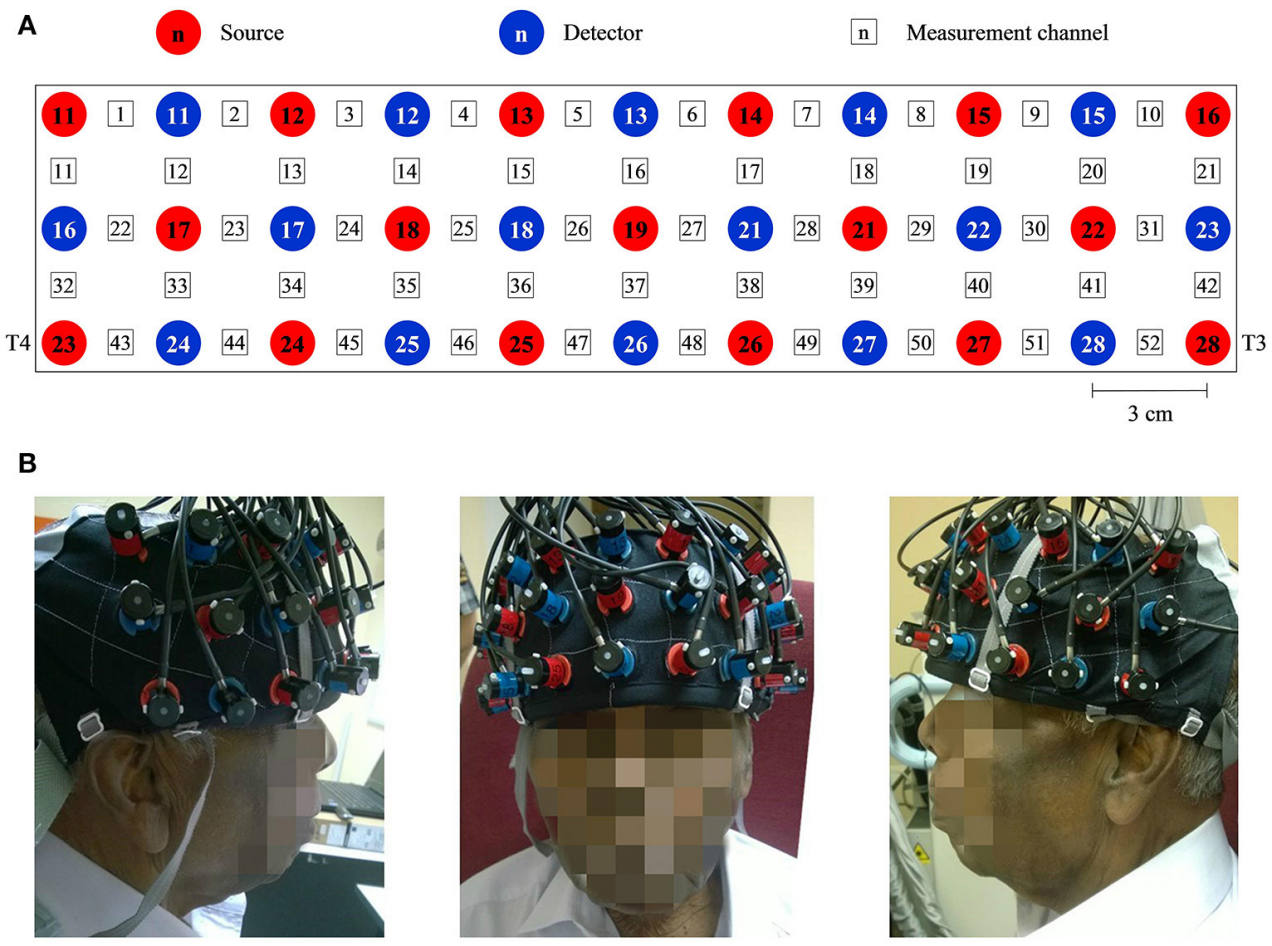
A system consisting of 52 measurement channels was used. **(A)** The probes were arranged to 3 × 11 layout. The source-detector distance was fixed at 3 cm and the space between pairs of source and detector was defined as a measurement channel. **(B)** One of the participants wearing the flexible head cap which housed the probes. Source no. 23 and 28 were positioned directly at T4 and T3 accordingly to the international 10–20 system. Consent was obtained from the individual for the publication of this image.

Throughout this study, a multichannel OT-R40 fNIRS topography system (Hitachi Medical Corporation, Japan) was employed to measure the brain activity at a sampling rate of 10 Hz. Changes in oxy-Hb and deoxy-Hb signals were measured in units of mM·mm. fNIRS has been reported to be more sensitive to gray matter when a larger source-detector separation (up to ~4.5 cm) is used, albeit at the expense of both spatial resolution and partial pathlength factors (Strangman et al., 2013). Taking everything into consideration, the source-detector distance was fixed at 3 cm, within the suggested optimal range for adult heads (3–3.5 cm) (Li et al., 2011). The midpoint between pairs of sources and the detector was defined as a measurement channel. The probes were arranged into a 3 × 11 layout (see Figure 1A) to form a total of 52 measurement channels that were sufficient to measure the entire PFC and part of the temporal cortex (see Figure 1B; Ishii-Takahashi et al., 2014; Takizawa et al., 2014). According to the international 10–20 system (Klem et al., 1999), source no. 23 and 28 were positioned directly at T4 and T3, respectively. The probes were attached to a flexible head cap, which was relatively easy, fast and convenient to wear. All channels were checked to ensure that the probes were in contact with the scalp. The entire set-up process took an average of <10 min.

### Task paradigm

**Figure 2:**
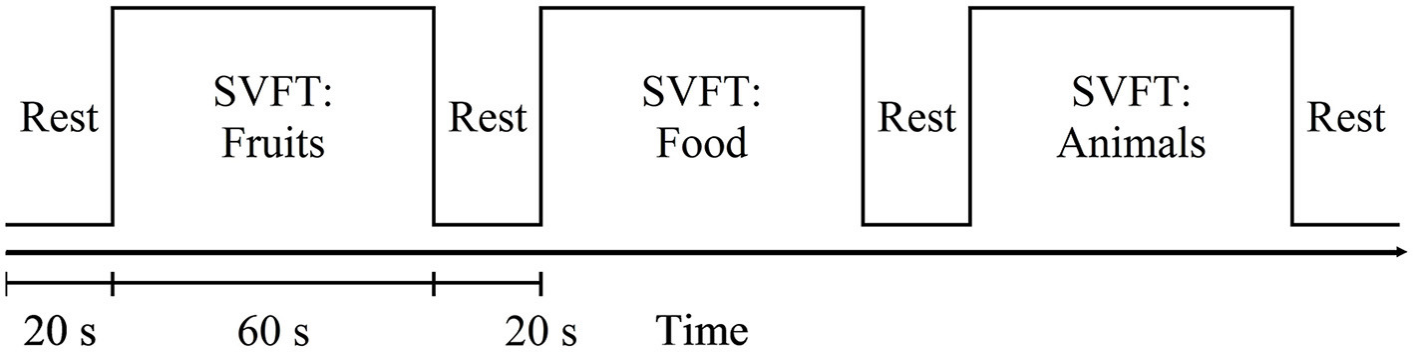
Schematic diagram of the measurement sequence: SVFT = semantic verbal fluency test. It started with a 20 s rest. Each category of SVFT was 60 s long and was followed by 20 s of rest.

Participants were seated comfortably in a working chair and were instructed to avoid movement and to place their hands on the armrests during the experiment. Standardized verbal instructions and explanations regarding the tasks were given in English. Prior to any new measurement, practice was given, allowing the participants to familiarize themselves with the experimental procedures. SVFT was selected as the cognitive activation task in this study. During fNIRS measurements, participants were instructed to provide as many words verbally as possible from a particular category (Fruits, Food, and Animals). The experimental session was preceded by 20 s of pre-task rest period. Each category lasted 60 s and was followed by 20 s of rest. Participants were told to avoid repeating the same word and they were asked to keep their eyes on the LCD screen for the entire task period, which lasted for a total of 260 s (see Figure 2).

### Data analysis

#### fNIRS data

Oxy-Hb was selected as the focus of measurements due to its sensitivity to task-associated changes (Sato et al., 2006; Cui, 2011). Probes that were not in good contact with the scalp may have resulted in rapid large changes (such as high amplitude spikes) in oxy-Hb signals. An fNIRS channel was considered to be “noisy” if there were very large spikes (changes in oxy-Hb larger than 0.5 mM·mm in amplitude) and all noisy channels were excluded from subsequent analyses. All data analyses were performed using a software Platform for Optical Topography Analysis Tools (Sutoko et al., 2016). A Butterworth bandpass filter with cut-off frequencies of 0.01–0.8 Hz was applied to remove instrumental or physiological noise (Luu and Chau, 2009).

**Figure 3:**
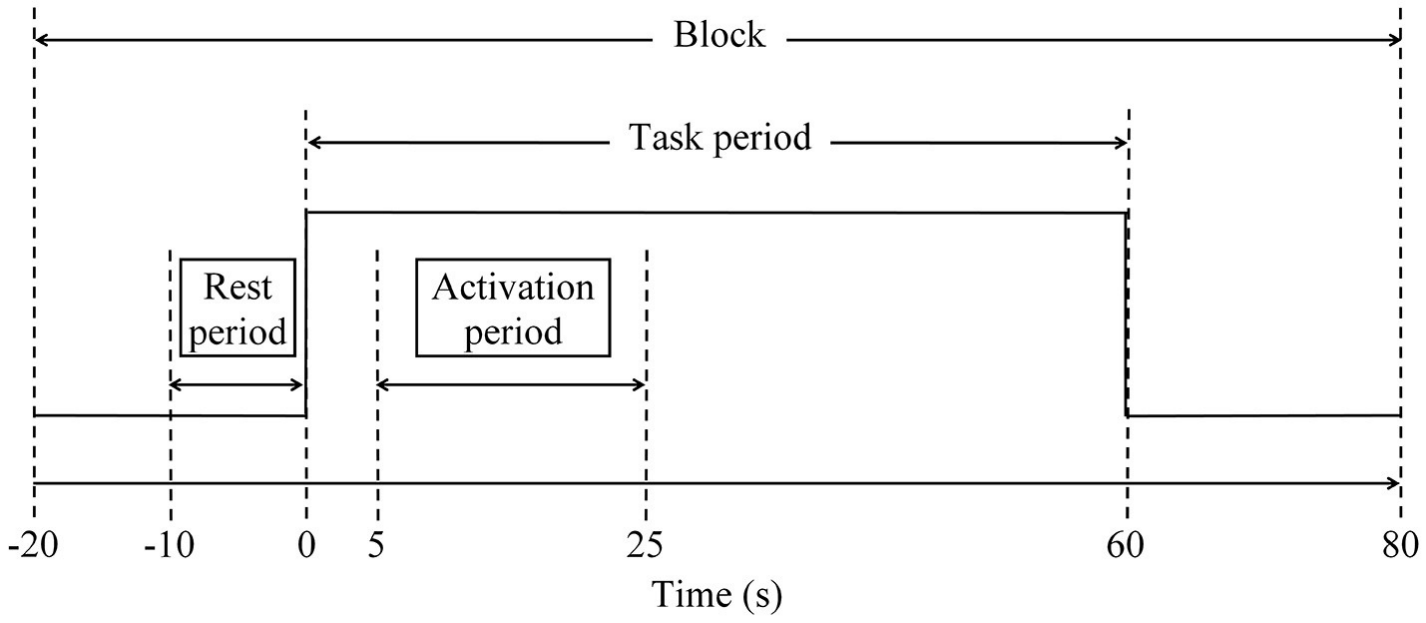
Schematic diagram of a block to facilitate analysis. Each block consisted of 20-s pre-task rest period, 60-s task period and 20-s post-task period. Three categories of SVFT can be represented as three 100-s blocks.

Three categories of SVFT were represented by three 100-s blocks of data for analysis. As discussed in the task paradigm, each block consisted of a 20-s pre-task rest period, a 60-s task period followed by a 20-s post-task period (see to Figure 3). A moving average filter with a window size of 50 data points (5 s) was applied to remove high frequency noise from the measured oxy-Hb signals (Ishii-Takahashi et al., 2014). With respect to the start of each block, the oxy-Hb signals were then baseline-corrected to a zero baseline. Blocks with a large spike noise in the oxy-Hb signal (changes in oxy-Hb larger than 0.5 mM·mm in amplitude) were excluded from further analyses. Subsequently, fNIRS signals from each channel were meaned over all the remaining blocks so that each subject should have had only one average fNIRS signal per channel. Subsequent analyses were performed using these average fNIRS signals. Previous fNIRS studies have suggested that earliest activation starts from 5 s after task onset (Sato et al., 2006) and sharp increases in activation are often observed at around 5–10 s after onset (Maki et al., 1995). Therefore, the level of activation at each channel was determined for each individual using the percentage signal change, which was calculated using the following formula:

(1) $$\begin{array}{l} percent\;signal\;change\\ =\;\frac{oxy-Hb_{t\;=\;5:65\;\left(avg\right)}-\;oxy-Hb_{t\;=\;-10:0\;\left(avg\right)}}{\left|oxy-Hb_{t\;=\;-10:0\;\left(avg\right)}\right|}\;\times 100\% \end{array}$$

where *oxy* − *Hb*_*t* = 5:65 (*avg*)_ is the average oxy-Hb signal during the task period, after accounting for hemodynamic delay, and *oxy* − *Hb*_*t* = −10:0 (*avg*)_ is the average oxy-Hb signal during the rest period (−10–0 s of the pre-task rest period; see Figure 3). Channels located in the PFC that showed a percentage signal change of larger than 50% were empirically considered to be activated and were further divided into left and right PFC. Hence, it was possible for each subject to have a different number of activated channels, but they were all within the regions of interest (the left and right PFC).

Due to hemodynamic delay, the period between 5 and 25 s after task onset was defined as the activation period (see Figure 3). The activation signal was defined as the difference between the average oxy-Hb signal during the activation period and during the rest period. For each participant, the mean activation signal in the left and right PFC was calculated by averaging the signals obtained in activated measurement channels located in the left and right PFC, respectively. Based on the mean activation signals, for both the left and right PFC, the time taken to achieve the activation level was determined and the slope in the first 5 s after task onset was calculated using the following formula:

(2) $$\begin{array}{l} slope=\;\frac{oxy-Hb_{left\;PFC/right\;PFC}^{t=5}-\;oxy-Hb_{left\;PFC/right\;PFC}^{t=0}}{5}\;\\ \times \;\frac{\text{mM}\cdot \text{mm}}{\text{s}} \end{array}$$

where $oxy-Hb_{left\;PFC/right\;PFC}^{t=0}$ and $oxy-Hb_{leftPFC/rightPFC}^{t=5}$ denote the mean activation signals in the left or right PFC at the onset of the task and 5 s after the onset of the task, respectively.

The number of activated measurement channels, the mean activation signals in both the left and right PFC, and the time taken to achieve the activation level and the slope in the first 5 s after task onset were then statistically assessed.

#### Statistics

Differences between all three groups in MMSE scores were first tested using the multiple non-parametric Mann–Whitney U tests without multiple testing correction, followed by Mann–Whitney U tests with Bonferroni-Holm correction. Both tests were repeated to assess whether there were any within-category group differences in the number of words generated in the three categories of SVFT. The Bonferroni-Holm correction would test each individual hypothesis in a sequential rejective manner at α/[*n* − *rank number of the pair* (*by degree of significance*) + 1], where α is the desired significance level (0.05) and *n* is the number of comparisons. fNIRS data that were statistically assessed included the number of activated measurement channels, the mean activation signals in both the left and right PFC, the time taken to achieve the activation level and the slope in the first 5 s after task onset. For each of these four fNIRS parameters, statistical significance was estimated using multiple comparisons between the three different groups at each category level, with two sets of independent two-sample *t*-tests—one without multiple testing correction and one with similar Bonferroni-Holm correction. Finally, to investigate the relationship between the MMSE score and various fNIRS parameters, correlation and simple linear regression analyses were performed using the MMSE score as a continuous independent variable.

## Results

### Sample characteristics

We excluded the data collected from one patient with moderate AD (CDR Score = 2) as well as two left-handed participants (one each from HC and MCI, as we were focusing only on right-handed participants). The final group of participants consisted of 31 HC and 30 patients (MCI: 12; mild AD: 18) matched for age, sex, education, and handedness. Demographic information about age, gender and education level was collected (see Table 1).

**Table 1:** Participants' demographic information and the pairwise Mann-Whitney U**-**test results.

	**HC (n = 31)**	**MCI (n = 12)**	**mild AD (n = 18)**	**Mann–Whitney U-test**		
				***p*****-value (*****r*****)**		
**Characteristic**	**Mean (SD)**	**Mean (SD)**	**Mean (SD)**	**HC vs. MCI**	**HC vs. mild AD**	**MCI vs. mild AD**
Age, years	72.6 (8.5)	73.1 (8.2)	74.7 (10.0)			
Gender, M/F	19/12	8/4	12/6			
Education level, P/S/T	3/18/10	1/7/4	3/12/3			
CDR rating	0	0.5	1			
MMSE score	28.7 (1.5)	26.0 (3.1)	21.2 (3.6)	0.0033^**^ (0.4485)	< 0.0001^**^ (0.7957)	0.0012^**^ (0.5896)
SVFT, words						
Fruits	13.6 (4.9)	9.0 (3.7)	5.7 (2.2)	0.0081^**^ (0.4036)	< 0.0001^**^ (0.6934)	0.0084^**^ (0.4809)
Food	14.6 (4.9)	10.2 (3.9)	6.3 (2.7)	0.0094^**^ (0.3961)	< 0.0001^**^ (0.7459)	0.0079^**^ (0.4851)
Animals	15.2 (3.7)	10.8 (5.7)	7.8 (3.0)	0.0217^**^ (0.3500)	< 0.0001^**^ (0.7304)	0.1669 (0.2524)

** *p < 0.05 with Bonferroni-Holm correction*.

*HC, healthy control; MCI, mild cognitive impairment; mild AD, mild Alzheimer's disease; r, effect size; SD, standard deviation; M, male; F, female; P, primary; S, secondary; T, tertiary*.

### Behavioral data

**Figure 4:**
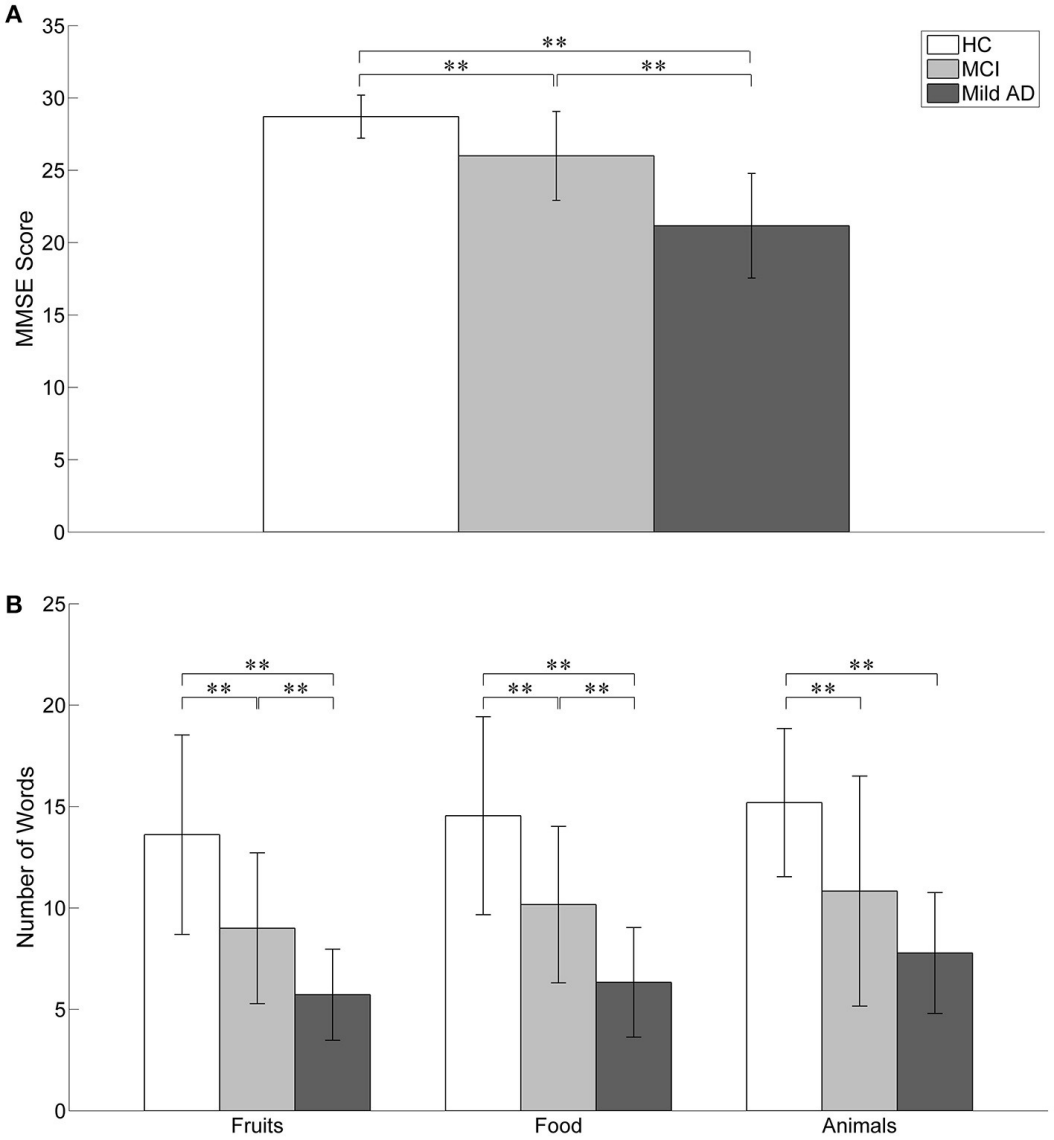
The behavioral results. Statistical analysis was performed used two-sample *t*-test. ^**^p < 0.05 with Bonferroni-Holm correction. The error bars represent the standard deviations. **(A)** The MMSE score of HC was significantly higher than MCI and mild AD. MCI also achieved significant higher MMSE score as compared to mild AD. **(B)** In the SVFT, the performance was measured by the number of words given. Significant group differences between all three groups were found in the number of words given in “Fruits” and “Food” categories. For “Animals” category, the number of words given by HC was significantly higher than mild AD. In comparison to MCI, HC produced more words but it was not statistically significant. The number of words given by MCI was also higher than mild AD but the difference was not significant.

The behavioral results (MMSE scores and the number of words generated in the three categories of SVFT) are summarized in Table 1 and illustrated in Figure 4. The number of comparisons for MMSE scores is three (for the three groups) and for SVFT is nine (3 groups × 3 categories). There was a significant difference in MMSE score between the groups (see Table 1 and Figure 4A; *p*: HC vs. MCI = 0.0033, HC vs. mild AD < 0.0001, MCI vs. mild AD = 0.0012). HC had the highest MMSE scores, followed by MCI, then mild AD. Referring to Figure 4B, significant group differences in the number of words given in the “Fruits” category (*p*: HC vs. MCI = 0.0081, HC vs. mild AD < 0.0001, MCI vs. mild AD = 0.0084) and the “Food” category (*p*: HC vs. MCI = 0.0094, HC vs. mild AD < 0.0001, MCI vs. mild AD = 0.0079) were found. For the “Animals” category, the number of words given by HC was significantly higher than for mild AD (*p* < 0.0001). In comparison to MCI, HC produced more words, but it was not statistically significant (*p* = 0.0217). The number of words given by MCI was also higher than mild AD, but the difference was not statistically significant (*p* = 0.1669).

### fNIRS data

**Figure 5:**
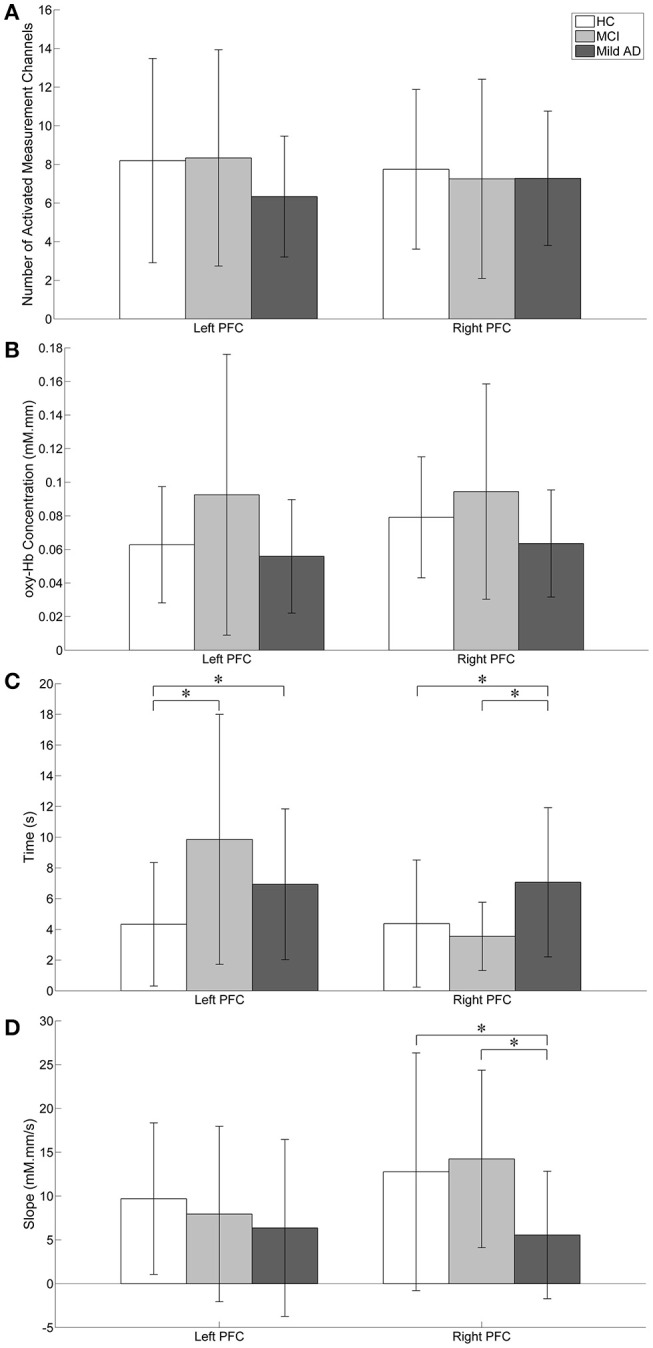
Visual representation of fNIRS data. Statistical analysis was performed used two-sample *t*-test. ^*^p < 0.05 without multiple testing correction. The error bars represent the standard deviations. **(A)** The number of activated measurement channels: *N*_*left PFC*/*right PFC*_; there were no significant differences between groups in both the left and right PFC. **(B)** oxy-Hb concentration change during the activation period: Δ*oxy* − *Hb*_*left PFC*/*right PFC*_; Higher activation was observed in MCI followed by HC while mild AD showed the least in both the left and right PFC. The right PFC was more activated than the left PFC in all groups. For both the left and right PFC, there were no significant group differences. **(C)** Time taken to achieve activation level: *t*_*left PFC*/*right PFC*_; HC's *t*_*left PFC*_ was significantly shorter than MCI. **(D)** Slope in the first 5 s after task onset: *m*_*left PFC*/*right PFC*_; in comparison to mild AD, MCI showed significantly steeper *m*_*right PFC*_.

To characterize the fNIRS responses, we derived the following parameters, as shown in Figure 5:

- the number of activated measurement channels, *N*_*left PFC*/*right PFC*_
- the change in oxy-Hb concentration during the activation period, Δ*oxy* − *Hb*_*left PFC*/*right PFC*_
- the time taken to achieve the activation level, *t*_*left PFC*/*right PFC*_
- the slope in the first 5 s after task onset, *m*_*left PFC*/*right PFC*_

**Figure 6:**
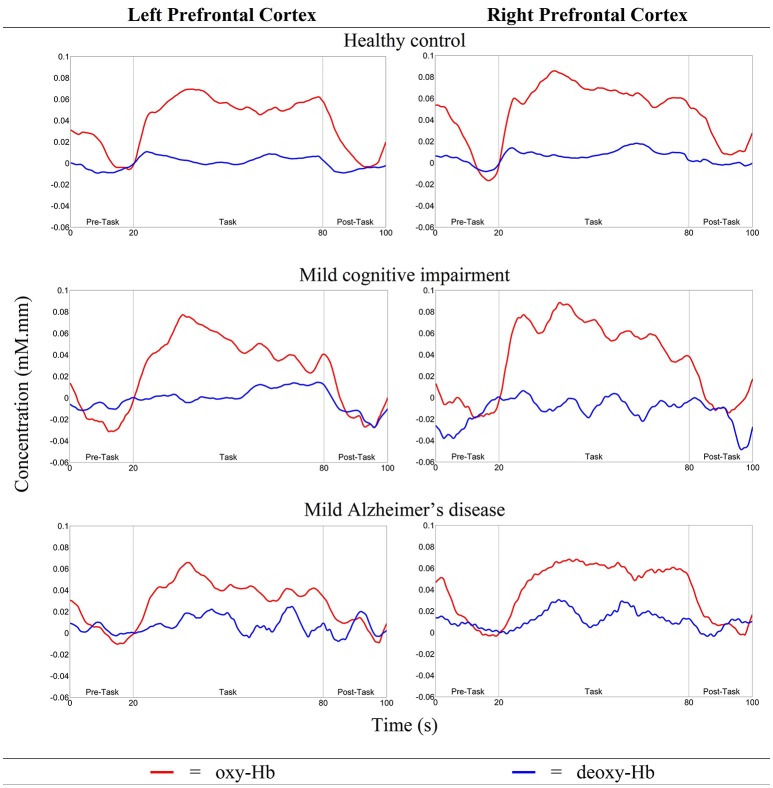
The overall oxy- and deoxy-Hb signals in the left and right PFC for all three groups.

As illustrated in Figure 5A, *N*_*left PFC*_ was found to be higher (but not significantly so) than *N*_*right PFC*_ in both HC and MCI. However, mild AD showed a higher *N*_*right PFC*_, compared to the *N*_*left PFC*_. The oxy-Hb signals measured in these activated measurement channels were then averaged across each group of participants to obtain an overall signal for the left and right PFC (see Figure 6).

Figure 5B shows the Δ*oxy* − *Hb*_*left PFC*/*right PFC*_ for all groups. The highest Δ*oxy* − *Hb*_*left PFC*_ was observed in MCI followed by HC, while mild AD showed the lowest Δ*oxy* − *Hb*_*left PFC*_. A similar trend was also observed in the right PFC. However, in both the left and right PFC there were no significant differences between all three groups with respect to the level of activation. Nevertheless, all three groups demonstrated a similar trend, with higher Δ*oxy* − *Hb*_*right PFC*_ compared to Δ*oxy* − *Hb*_*left PFC*_, although differences were not statistically significant.

The *t*_*left PFC*/*right PFC*_ was calculated and is illustrated in Figure 5C. When no multiple testing correction was used, the *t*_*left PFC*_ by HC was shorter than MCI (*p* = 0.0047) and mild AD (*p* = 0.0498). On the other hand, mild AD took a longer time than HC (*p* = 0.0469) and MCI (*p* = 0.0335) in the right PFC. The *m*_*left PFC*/*right PFC*_ was also determined (see Figure 5D). Both HC and MCI showed steeper *m*_*right PFC*_ when compared to mild AD (*p* = 0.0432 and 0.0107, respectively). In summary, HC used a shorter *t*_*left PFC*_ than MCI and mild AD, and mild AD took longer *t*_*right PFC*_ than HC and MCI, while HC and MCI demonstrated a steeper *m*_*right PFC*_ than mild AD. However, the results described above were not significant when a Bonferroni-Holm correction was applied (number of comparisons is 3 groups × 4 fNIRS parameters = 12).

**Figure 7:**
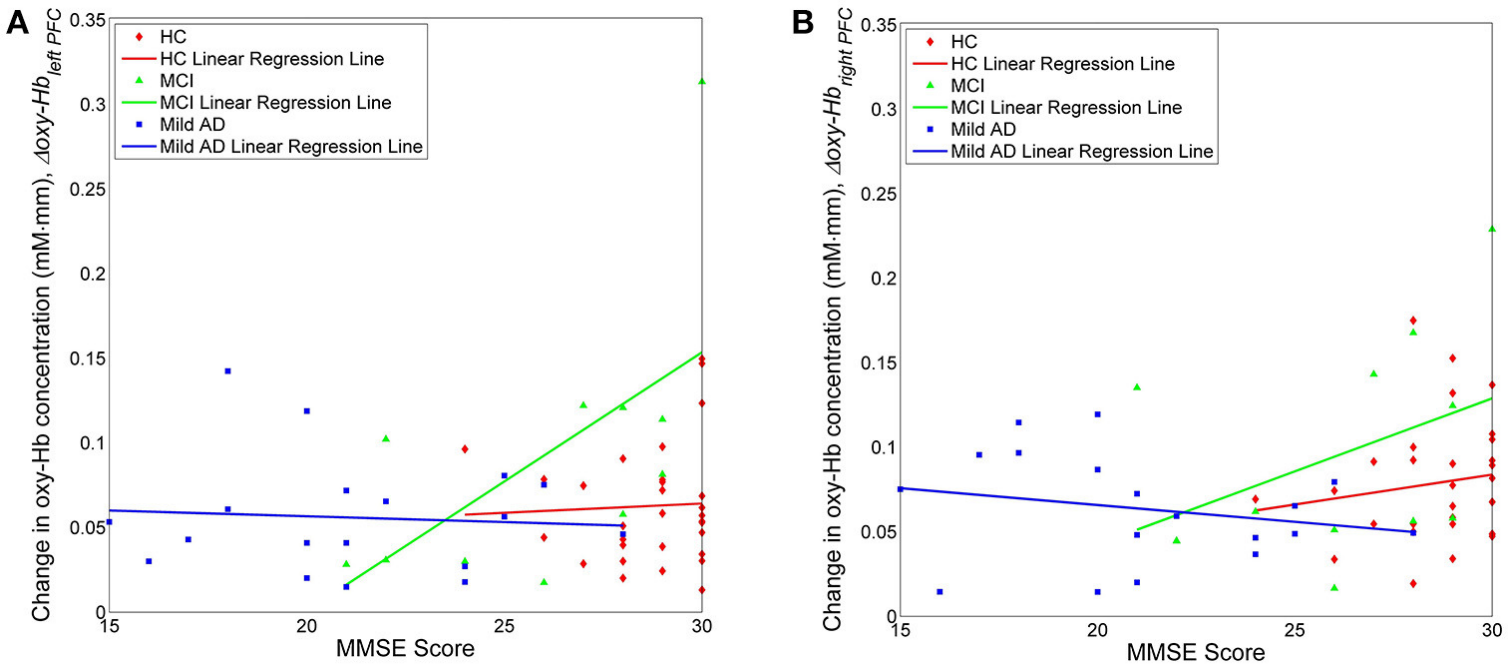
Change in oxygenation level in the left **(A)** and right **(B)** PFC for all three groups against clinical measure, i.e., MMSE score.

We also investigated, using simple linear regression, if there was any correlation between fNIRS parameters, i.e., *N*_*left PFC*/*right PFC*_, *N*_*left PFC*/*right PFC*_, *t*_*left PFC*/*right PFC*_ and *m*_*left PFC*/*right PFC*_, with behavioral data (MMSE scores). Interestingly, there was a moderate positive correlation (*R* ≥ 0.5) between one of the fNIRS parameters and the MMSE score in MCI, but not in the case of HC or mild AD. More specifically, in the left PFC, Δ*oxy* − *Hb*_*left PFC*_ was moderately correlated to the MMSE score (*R* = 0.5886), as illustrated in Figure 7; Table 2 summarizes the results.

**Table 2:** Pairwise correlation and simple linear regression results between fNIRS parameters, and MMSE score.

	**Left PFC**		**Right PFC**	
	***m***	**R**	***m***	**R**
**NUMBER OF ACTIVATED MEASUREMENT CHANNELS**,				
***N*_*left PFC*/*right PFC*_ vs. MMSE SCORE**				
HC	0.1919	0.0540	0.3868	0.1393
MCI	−0.1154	−0.0634	0.1731	0.1032
Mild AD	0.2517	0.2914	0.0412	0.0429
**oxy-Hb CONCENTRATION CHANGE DURING THE ACTIVATION PERIOD**,				
**Δ*oxy*−*Hb*_*left PFC*/*right PFC*_ vs. MMSE SCORE**				
HC	0.0010	0.0478	0.0035	0.1467
MCI	0.0152	0.5886	0.0086	0.4144
Mild AD	−0.0006	−0.0732	−0.0019	−0.2258
**TIME TAKEN TO ACHIEVE 25% OF PEAK ACTIVATION LEVEL**,				
***t*_*left PFC*/*right PFC*_ vs. MMSE Score**				
HC	−0.8478	−0.3394	−0.8567	−0.3713
MCI	−0.4644	−0.3340	0.4153	0.3143
Mild AD	0.0961	0.0729	−0.3606	−0.2733
**SLOPE IN THE FIRST 5 S AFTER TASK ONSET**,				
***m*_*left PFC*/*right PFC*_ vs. MMSE SCORE**				
HC	0.0008	0.2040	0.0020	0.2926
MCI	0.0007	0.2028	−0.0001	−0.0394
Mild AD	0.0000	−0.0026	0.0004	0.2663

*m, slope of the linear regression; R, correlation coefficient between the two parameters; HC, healthy control; MCI, mild cognitive impairment; mild AD, mild Alzheimer's disease*.

Finally, we noticed a large degree of inter-subject variation in fNIRS parameters, as shown in Figure 5. Such high variance not only makes the small group differences hard to distinguish statistically, but also results in a large overlap between the data from all three groups.

## Discussion

### Behavioral data

This study was designed to investigate the differences in prefrontal oxygenation between normal cognitive aging, MCI and mild AD using fNIRS. On a behavioral level, HC performed better than MCI, followed by mild AD in all three categories of SVFT, except for with the “Animals” category between HC and MCI; here, no significant difference was found between MCI and mild AD. We also observed that participants, regardless of study group, were relatively faster in providing responses during the “Animals” category compared to the “Food” and “Fruits” categories. This finding might be due to all three groups being more familiar with the names of animals. This has been reported in previous studies, which have shown that naming is influenced by item frequency and familiarity (Patterson and Hodges, 1992; Lambon Ralph et al., 1998). It is possible that this difference between categories may be related to the varying degree of distinctive features among category members (Moss et al., 2002). This will not be elaborated upon further here, as it is not the main focus of the present study. Overall, these results were consistent with past studies that have utilized SVFT in various neuroimaging modalities (Fallgatter et al., 1997; Heinzel et al., 2013; Wagner et al., 2014; Yeung et al., 2016). This suggests that the SVFT is a reliable cortical activation task to be used in conjunction with fNIRS measurements. Here, it was observed that patients diagnosed with a greater degree of dementia, i.e., with higher CDR scores, gave repeated words more frequently. They had a tendency to forget which words they had already given, an attribute of a deteriorated right PFC where the working memory is for monitoring, or keeping immediate information on-line during tasks (Hayama and Rugg, 2009). The number of repetitions in SVFT and such attributes will not be discussed further here.

### fNIRS data

#### *t*_*left PFC*/*right PFC*_ and *m*_*left PFC*/*right PFC*_

The results suggested that there were two fNIRS parameters that were significantly different between groups when the subjects were engaged in SVFT, only when no multiple testing correction was used. The first parameter is the *t*_*left PFC*/*right PFC*_. In the left PFC, HC took a shorter time in achieving the targeted activation level, compared to patients with MCI and mild AD. However, contrasting with the belief that MCI would have a faster activation than mild AD, patients with mild AD actually took a shorter time. Conversely, with respect to the right PFC, MCI demonstrated the shortest time taken, followed by HC and mild AD. This is suggestive of the faster hemodynamic response in the right PFC of the MCI possibly being a compensatory response for the loss of the left PFC (Yeung et al., 2016). Taken separately, the poorer performance in SVFT and smaller activation in conjunction with the shorter time taken for left PFC activation in patients with mild AD might suggest that the compensatory mechanism is compromised. The second parameter examined was the *m*_*left PFC*/*right PFC*_. Experimental results showed that the *m*_*right PFC*_ was significantly greater in MCI compared to mild AD, further suggesting its importance to describing compensatory mechanisms. While the Bonferroni-Holm correction demonstrated that there were no significant differences, it is worthwhile explaining here the underlying mechanisms for these results.

Two possible explanations have been proposed for the underlying mechanism. The first explanation was consistent with that of the scaffolding theory of aging and cognition, in which additional circuitry is recruited to support declining brain function that has become inefficient (Park and Bischof, 2013). This is commonly manifested in older adults that show increased contralateral right PFC recruitment for both working memory and episodic encoding (Reuter-Lorenz et al., 2000; Cabeza et al., 2002), which is consistent with our results. Such bilateral activation may be a form of interhemispheric interaction that has been claimed to be vital in neural compensatory mechanisms (Banich, 1998; Cabeza, 2002). Additionally, the results presented here imply that compensation and neuroplasticity might be present in the PFC of MCI, but not in mild AD. It has also been suggested that such compensatory ability might be reduced or lost in the progression of MCI toward AD, as neurodegeneration suffered by AD patients is severe enough to halt natural compensation (Clement et al., 2013). This is supportive of the results presented here, where patients with mild AD tended to forget which words they had already given added to the lack of activation of their right PFC, which is responsible for monitoring the immediate information during a task (Hayama and Rugg, 2009). This is further supported as the right PFC is specifically involved in semantic aspects of lexico-semantic processing (Joanette and Goulet, 1986).

We propose further, that the additional right PFC involvement and poorer memory performance can be explained using the inhibitory hypothesis. According to the inhibitory hypothesis, the non-dominant right PFC is normally suppressed by its dominant contra-lateral counterpart (Cox et al., 2015). Such transcallosal inhibition might be impaired with left PFC or anterior corpus callosum atrophy, which might result in extra recruitment of the right PFC. It has been found that additional non-dominant right PFC activity might reflect age-related changes in the brain and has been reported to be negatively correlated with memory performance (de Chastelaine et al., 2011). However, it has also been suggested that such disinhibition could reflect an attempted compensatory process, which is insufficient to fully compensate for age-related neurodegeneration. Our results point more toward the first explanation as we found that MCI, with right PFC activation relatively higher than both HC and mild AD, actually performed better than the latter in the SVFT. Hence, we suggest that the identification of a compensatory role for the right PFC might offer a potential target area for neurorehabilitation (Cotelli et al., 2008). It is expected that in the future the residual plasticity in the right PFC of cognitively impaired patients, particularly those with MCI, might be effectively harnessed by neurorehabilitation and other interventional techniques. At this juncture, it is necessary to examine other parameters to identify differences in task-related activities across different populations.

#### *N*_*left PFC*/*right PFC*_

Considering the number of activated fNIRS channels, there were no significant differences between both the right and left PFC for all three groups. The left PFC, which is responsible for semantic memory, was activated in the SVFT, as expected (Grossman et al., 2002). The activation of the right PFC, however, may be suggestive of participants being engaged in object imagery prior to recalling names during the task. In addition, it might also indicate ongoing monitoring of semantic information (Hayama and Rugg, 2009); the episodic memory located in the right PFC was engaged to ensure that participants did not repeat names during the task. As compared with HC, a smaller region of right PFC activation was found in the other two groups (MCI and mild AD). This could indicate the lack of a monitoring process, thus partially explaining poorer performance in SVFT in these groups compared to HC. However, this cannot explain the differences in SVFT performances between MCI and mild AD.

#### Δ*oxy*−*Hb*_*left PFC*/*right PFC*_

MCI demonstrated the greatest oxygenation levels of PFC activation, in both the left and right hemispheres, during SVFT, followed by HC, and mild AD. Despite not being statistically significant, this result might be of clinical significance. The result is in agreement with previous studies, utilizing various neuroimaging modalities, which have demonstrated similar findings (Johnson et al., 2000; Arai et al., 2006; Sperling, 2007; Driscoll et al., 2009; Woodard et al., 2009). As the dorsolateral PFC has been suggested to be associated with compensatory mechanisms (Erickson et al., 2007), the brain region-specific hyperactivation and hypoactivation observed in MCI and mild AD, respectively, might indicate the presence of neural compensation in the former, and the inability to compensate in the latter (Prvulovic et al., 2005; Clement and Belleville, 2012). The differences in the level of activation across groups might also explain the differences in performance in SVFT, particularly in the left hemisphere (Fallgatter et al., 1997; Arai et al., 2006; Herrmann et al., 2008). However, contrary to current opinion, in the present study activation in the right PFC was greater compared to the left PFC, and was consistent between all the groups. This might be for the following reasons: (a) the activation of right PFC in monitoring cognitive processes during SVFT might implicate a greater role compared to the left PFC; (b) the difference might be due to methodological differences, including the differences in recruitment strategies, brain region investigated and variations in research design and measurements used to describe the outcome. More specifically, the variations in research design refer to different assessment tools, inclusion and exclusion criteria (Fallgatter et al., 1997; Yeung et al., 2016), the numbers of groups (e.g., between normal aging and AD; Herrmann et al., 2008) or between normal aging and MCI (Yeung et al., 2016), and the focus on a brain region (e.g., frontal and parietal lobe). Similarly, differences in the outcomes measured might exert a significant impact on the results, such as separate analyses of oxy-Hb and deoxy-Hb (Herrmann et al., 2008) and different baseline-corrected values (Fallgatter et al., 1997). The high variance observed here might be due to placement of the probes i.e., the position of the optodes relative to the skin (Strangman et al., 2003). Inter-subject anatomical variability, such as the thicknesses of the skull and the cerebrospinal fluid layers could have also caused the large variation in the fNIRS measurements (Okada and Delpy, 2003).

#### Moderate positive correlation between Δ*oxy*−*Hb*_*left PFC*_ and MMSE score in MCI

Finally, we propose an explanation for the moderately positive (*R* = 0.5886) linear relationship between the oxygenation level in the left PFC and the MMSE score, which was found only in MCI subjects, but not in HC and mild AD. This result reflects the fact that increasing activation actually contributed to the cognitive status in MCI, while both HC and mild AD did not show a similar trend. We suggest that in HC, the behavioral ceiling effect might have been achieved, as represented by the maximum score in MMSE (30) and this does not necessarily imply the presence of a parallel ceiling in brain activation (continuous increases in left PFC activation) (Hagenbeek et al., 2007). This might also indicate that the neural network is intact in HC. In addition, MMSE might be a relatively easy task for HC and the maximum score of 30 points may, therefore, not be a sensitive measure of cognitive status. MMSE is not designed to measure the cognitive ability of a healthy person. However, cognitive status in MCI is well below the ceiling. The pathogenesis of AD is characterized pathologically by brain accumulation of amyloid β-protein (Aβ) in the early stages (Jack et al., 2013) and Aβ is thought to be the cause of neuronal dysfunction in AD (Palop and Mucke, 2010), which necessitates neural activity. Previous studies have reported that, relative to younger people or older people without Aβ, both cognitively normal older people with Aβ deposition (Mormino et al., 2012) and MCI patients (Dickerson et al., 2004) exhibit higher neuronal activity during cognitive task performance. This phenomenon might be evidence of functional compensation keeping older people with Aβ and MCI patients cognitively stable. In agreement with these results, the increase in oxygenation levels might represent an attempted compensatory response, and hence it is proportionate to the improvement in cognitive status in MCI. However, it is not possible to draw any conclusions with respect to the interpretation due to the small MCI sample size here. Meanwhile, for mild AD, no linear relationship was found between the oxygenation levels in the left PFC and the MMSE score. The degree of Aβ deposition in the brain might reduce neural efficiency, which eventually causes progression to a more severe stage of AD (Landau et al., 2012). In such a situation, it is possible that patients eventually decline cognitively as any compensatory ability has been compromised (O'Brien et al., 2010). So, when patients progress into mild AD, their neural compensation ability might have been weakened or the neural networks might have been compromised to the point where higher oxygenation levels coupled with compensatory mechanisms are no longer enough to maintain cognitive status, unlike with MCI. Although the explanations above might fit with the observations, it is important to note that various non-neural factors might confound such interpretations; disrupted neurovascular coupling that is associated with pathological conditions e.g., aging and disease (Buckner et al., 2000; D'Esposito et al., 2003) is one of the many factors. Other factors include alterations in perfusion and metabolism (El Fakhri et al., 2003), and vascular physiology (Mueggler et al., 2002). It has also been reported that employing different verbal memory strategies led to different patterns of cortical activation (Logan et al., 2002). A final factor that might have influenced PFC activation is the administration of medication e.g., cholinergic stimulation (Rombouts et al., 2002) and donepezil (McGeown et al., 2010).

### Limitation

Subtypes of MCI need to be considered: a clinical presentation with memory impairment is characterized as amnestic MCI (aMCI), whereas the absence of memory impairment with the presence of impairment in one or more non-memory cognitive domains is characterized as non-amnestic MCI (naMCI). Furthermore, these subtypes can be further narrowed down into single and multi-domain impairments. It has been suggested that aMCI has a higher likelihood of progressing into AD, while naMCI is prone to developing into non-AD dementia (Petersen et al., 2009). Since the present study accessed a relatively small number of MCI patients, no attempt was made to exclude patients on the basis of other comorbidities. To substantiate the findings, research with a larger sample size might help ensure that participants with secondary comorbidities can be excluded. In addition, such a study could ensure that participants with different subtypes of MCI are assessed separately.

## Conclusion

It was found that HC took a shorter time to achieve the targeted activation level in the left PFC compared to MCI and mild AD, while mild AD took a longer time than HC and MCI in the right PFC. In addition, a steeper slope of activation was found in the right PFC of patients with MCI compared to HC and mild AD. The right PFC was particularly recruited in compensatory activity, which could be explained by the scaffolding theory of aging and cognition, and the inhibitory theory. Our results demonstrated, by using fNIRS, that compensation and neuroplasticity in the form of hyperactivation might be present in the PFC of MCI, but not in mild AD. Compensatory mechanisms might, therefore, have been compromised in mild AD. Time taken and the slope of activation were identified as key parameters of neuronal compensatory mechanisms, although the results presented here were not statistically significant after Bonferroni-Holm corrections. Future studies should look at these parameters individually. A moderately positive correlation between the oxygenation level in the left PFC and MMSE score was also found uniquely in MCI subjects. Longitudinal studies would be helpful in confirming whether task-elicited hyperactivation in MCI and hypoactivation in mild AD do indeed reflect the presence of compensatory mechanisms and the inability to compensate, respectively. If they do, future studies with a larger sample size could be directed toward investigating these fNIRS parameters as potential prognostic biomarkers of MCI and mild AD progression.

## Author contributions

TT, EE, and SS designed the study. KY, WU, NN, PC, SC, and HY acquired the data. KY, WU, MK, and TT analyzed the data. KY, WU, and TT wrote the article, which all authors reviewed and approved for publication.

### Conflict of interest statement

The authors declare that the research was conducted in the absence of any commercial or financial relationships that could be construed as a potential conflict of interest.

## Abbreviations

AD: Alzheimer's disease
aMCI: amnestic mild cognitive impairment
Aβ: amyloid β-protein
CDR: clinical dementia rating
deoxy-Hb: deoxygenated-hemoglobin
fMRI: functional magnetic resonance imaging
fNIRS: functional near-infrared spectroscopy
HC: healthy controls
MCI: Mild cognitive impairment
MMSE: mini-mental state examination
MRI: magnetic resonance imaging
naMCI: Non-amnesic mild cognitive impairment
oxy-Hb: oxygenated-hemoglobin
PFC: prefrontal cortex
SVFT: semantic verbal fluency task
*N*_*left PFC*/*right PFC*_: the number of activated measurement channels
Δ*oxy*−*Hb*_*left PFC*/*right PFC*_: the change in oxy-Hb concentration during the activation period
*t*_*left PFC*/*right PFC*_: the time taken to achieve activation level
*m*_*left PFC*/*right PFC*_: the slope in the first 5 s after task onset.
